# Plants for making wooden bowls and related traditional knowledge in the Gyirong Valley, Tibet, China

**DOI:** 10.1186/s13002-022-00514-y

**Published:** 2022-03-10

**Authors:** Xiao-Yong Ding, Chang-An Guo, Hua-Bin Hu, Yu-Hua Wang

**Affiliations:** 1 Yunnan Key Laboratory for Wild Plant Resources, Kunming Institute of Botany, Chinese Academy of Sciences, Kunming, China; 2 University of Chinese Academy of Sciences, Beijing, China; 3 CAS Key Laboratory of Tropical Plant Resources and Sustainable Use, Xishuangbanna Tropical Botanical Garden, Chinese Academy of Sciences, Mengla, 666303 Yunnan China

**Keywords:** Tibetan, Traditional handicraft, Wooden bowl, Ethnobotany, Governance

## Abstract

**Background:**

The wooden bowl is an important symbol of the Tibetan cultures, yet, in China, little has been documented regarding the raw materials used to make these items as well as their cultural significance in Tibet. This study explores the ethnobotanical uses of plants used to make wooden bowls to understand their sustainability, cultural significance, and current status of related traditional knowledge in Gyirong Town, which is one of the most famous places for wooden bowl making.

**Materials and methods:**

Between 2019 and 2021, key informant interviews, semi-structured interviews, and participatory observations were used to conduct ethnobotanical field surveys in Gyirong Valley. The field work was performed with the assistance of local guides. In this study, we utilized a use-report (UR) to reflect the number of mentions of a species by locals.

**Results:**

Our results show that 16 different plants are used during the wooden bowl making process, of which nine are used as raw materials, three for dyeing, and four for varnishing. Although communities rely heavily on these plants, good management and collection methods were observed. We also documented the use of *Fallopia denticulata* as a red dye and four species of *Impatiens* as wood varnishes for the first time.

**Conclusion:**

The wooden bowl craftsmen and their housewives have a wealth of traditional knowledge of using plants to make wooden bowls in Gyirong Town. And the wooden bowls are now also offering benefits to the locals as well. The government and local people are committed to the protection and development of traditional knowledge related to wooden bowls, and this knowledge maintains a healthy degree of vitality. This research can provide insights into the vitality of traditional handicrafts that are facing challenges and promote their protection.

## Background

The technique, artistic value and cultural connotation contained in handicraft products are all important aspects of intangible cultural heritage [1]. The handicraft industry also plays a vital role in generating income and employment [2, 3] and is recognized as a tool for poverty reduction worldwide. Indeed, in many developing countries, the handicraft industry is an important income sector second only to agriculture [4]; however, the handicraft industry faces some challenges, such as industrialization and globalization [5]. Moreover, due to the lack of sufficient data on handicraft practices, this sector remains at a disadvantage [5, 6], which hinders protection measures [7].

As an important representative of China’s intangible cultural heritage, traditional handicrafts of China are facing the risk of being lost due to the influence of modernization and industrialization [1]. Many plants are used as raw materials or auxiliary materials in the process of traditional handicraft making. Several ethnobotanical studies consider traditional handicrafts of use within their results, at the same time there are many anthropological and historical documents that mention them. A group of scholars from Beijing documented the traditional bamboo weaving plants and related knowledge of the Lhoba people in Milin, Tibet and the Miao people in Sanshui, Guizhou [8, 9]. And bamboo weaving has played a role in poverty alleviation in some place [8]. Kang et al. documented 84 wooden plant species for variable purpose in the Qinling mountains, China [10]. And some valuable species were usually selected use. Some plants that dye handicrafts (mostly cloth) were also documented [8, 11, 12]. The Yi people living in Liangshan, Sichuan, use *Oxalis corniculata* to scrub, de-tarnish, and polish the silver jewelry of traditional attire [13]. In China, wooden handicrafts are usually coated with lacquer produced by *Toxicodendron vernicifluum* to achieve aesthetics and protection [14].

Tibetans are one of 56 ethnic groups in China and have lived in the Qinghai-Tibet Plateau region for generations. In history, Tibet has three cultural regions (Kham, Ü-Tsang, and Amdo Tibet) [15]. The use of wooden bowls by Tibetans has a long history and is one of the necessities of Tibetan life, now an important symbol of Tibetan culture [16]. These wooden bowls made in many places, but regions in Cuo na, Jia cha, Gyirong, and Cha yu are particularly well known for their bowl-making skills. The craftsmanship in Gyirong and Cha yu wooden bowls has led to their recognition as a type of regional intangible cultural heritage of Xizang [17]. Wooden bowls are an indispensable part of Tibetan life and an important source of income for local Tibetans. Some studies mentioned the local names of the plant used to make the wooden bowl, while specific scientific names have not been documented [18, 19]. Huang describes that the coloring and lacquering of Tibetan wooden bowls generally involve the juice of “jia-yu-cao” as dye, which grows in mountainous areas [19].

The Tibetans speak the Ü-Tsang dialect in Gyirong Valley, and they are famous in the nearby area for processing wooden bowls. However, there are no listed plants for which data have been published for use as wooden handicrafts, wood coloring, and wood varnish. Building on this sparse information, we aimed to (1) document the traditional Tibetan plants and knowledge of wooden bowl makings in Gyirong Town, (2) explore the cultural significance of these plants in Tibetan traditional customs, and (3) explore whether there has a change in the utilization of plants used to make wooden bowls? Compared to the past, what changes happened to? (4) What is the local Tibetan traditional way of collecting and managing the plants for making wooden bowls?

## Materials and methods

### Study area

**Fig. 1:**
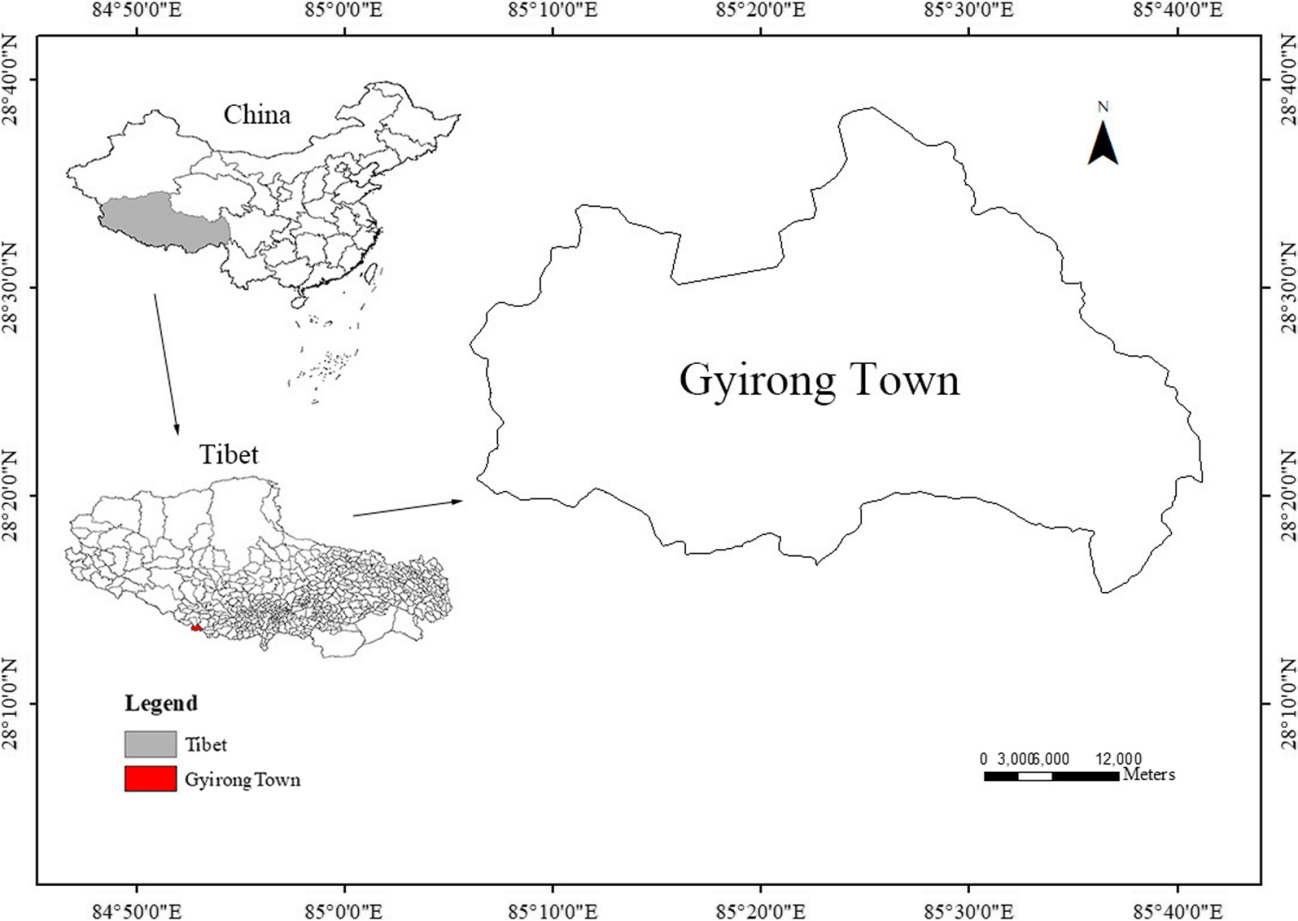
Study area: Gyirong Town, Tibet, China

Gyirong Town is known as the “Back Garden of the Himalayas” and is located in the core area of the Everest National Nature Reserve to the south of Shigatse City in the Tibet Autonomous Region of China (Fig. 1). The total population of the town was 3946 in 2017, and the main ethnic group is Tibetan. In Daman Village of Gyirong, there are still more than 200 Daman people. Their main production practice is to produce iron tools and sell them to the surrounding Tibetans, and they don't know how to make wooden bowls. This area has a subtropical monsoon climate with an annual average temperature of 10–13 °C (temperatures during the warmest months exceed 18 °C), an annual precipitation of 230–370 mm, and more than 200 frost-free days annually. Gyirong Town is dominated by low altitude forest dominated by *Pinus wallichiana*, *P. roxburghii*, *Picea smithiana*, *Tsuga dumosa*, *Betula utilis*, *Abies spectabilis*, *Sabina chinensis*, and *Cuculus* spp. This rich forest resource provides sufficient raw materials for traditional wooden bowl makings. The work report of the Gyirong Town People’s Government from 2016 to 2020 pointed out that during this period, Gyirong Town focused on carrying out more than 1500 forest fire prevention patrols. At the same time, it strengthened the management of handicraft and gathering industries to prevent the occurrence of random logging and digging. The traditional production practices of the Gyirong Tibetans are agriculture and grazing. The main crops are *Hordeum vulgare*, *Solanum tuberosum*, *Fagopyrum tataricum*, rapeseed. The traditional diet of the Tibetans in Gyirong is mainly tsampa, dairy products, and butter tea.

The Gyirong Valley has been an important communication channel between China and South Asian countries since ancient times. It can be said that the Valley has filled half of the history of Tibet. In 633 AD, Princess Chizun of Nepal entered Tibet from here when she married Tubo Zanpu Songtsen Gampo. During the Tang Dynasty of China, there was the official route of ancient Chinese envoys to South Asian countries. In 789 AD, Gyirong has become an important channel for exchanges and trade between Tibet and Nepal [20]. Therefore, Indian and Nepalese styles can still be observed in some temple buildings. Until now, the Tibetan of Gyirong Valley still maintains the traditional customs of transnational trade and intermarriage.

### Data collection

Between 2019 and 2021, key informant interviews, semi-structured interviews, and participatory observations were used to conduct ethnobotanical field surveys. To help understand the traditional knowledges of different stakeholders associated with Gyirong wooden bowls, three students and 20 housewives from the traditional handicraft families, 24 craftsmen (also a merchant, as the wooden bowls are sold directly from home to tourists) and four wooden bowl merchants who only sell wooden bowls and do not process were selected as key informants. The age of the informants ranged from 19 to 87. The “snowballing” method was used to identify the wooden bowl craftsmen, and random interviews were conducted of the students, housewives and merchants. A total of 51 informants (27 men and 24 women) were interviewed (Table 1), all of whom provided informed consent before the interviews. Informants of different genders and ages were selected. Due to relatively poor levels of education, most local people, especially community elders, could not communicate fluently in Chinese. Therefore, fieldwork was conducted with the assistance of local guides. The key informant and semi-structured interviews were conducted based on the following questions: (1) What tree can be used to make wooden bowls? (2) Which tree is the best? and why? (3) What plant do you use to dye the wooden bowls? (4) What plant do you use to varnish the wooden bowls? And why? (5) Do these plants or wooden bowls have any special meaning or symbolism? (6) has there been a change in the utilization of plants used to make wooden bowls ( such as the plant species, the parts used)? Compared to the past, what changes happened to? During the semi-structured interview, after asking the set questions, we also ask about management, selection, historical changes, and other relevant information according to the actual interview situation. The basic information of the plants, such as the vernacular name, the use part, and the use method, was documented during the semi-structured interviews.

**Table 1:** Distribution according to gender, age and specialty of the informants interviewed in Gyirong Town, Tibet, China

Characteristics	Number	Percentage (%)
*Communities*		
Gyirong town	51	100
*Gender*		
Man	27	53
Woman	24	47
*Age*		
19–30	7	13.7
31–40	6	11.8
41–50	16	31.4
51–60	10	19.6
61–70	9	17.6
71–87	3	5.9
*Occupation*		
Student	3	5.9
Housewife	20	39.2
Craftsman	24	47.1
Merchant	4	7.8

During the field surveys, with the help of key informants and local guides, we collected plant specimens according to the principle that one venecular name corresponds to one plant specimen. Permissions to take samples were always obtained from the informants and relevant local community departments. The specimens were identified and preserved in the herbarium of the Kunming Institute of Botany, Chinese Academy of Sciences (KUN), and the attributed scientific names were checked using “The Plant List.” [21]. As Gyirong Town is located on the Chinese border and the scope of activities during the COVID-19 pandemic is restricted, we did not collect a voucher specimen of *long-xin*. But according to the information described by the informants: this tree is very similar to *tang-xin* (*P. wallichiana*) and grows in lower altitude areas, so we speculate that *long-xin* may be the dominant species (*P. roxburghii*) in the low-altitude vegetation of Gyirong Valley. Except for this species, specimens of other species are collected and kept in the Kunming Herbarium.

### Data analysis

We adopted the use report (UR) as ethnobotanical index. A use report (UR) is the specific use of a species cited by an informant [22]. The number of UR can reflect the number of mentions of a species by locals.

## Results and discussion

### Plants used for manufacture wooden bowls in the different stages

In total, this study documented 16 plants belonging to 11 families and 13 genera being used at different stages of Gyirong wooden bowl processing (Table 2). Their vernacular names, parts used and use reports for all species mentioned by different gender were documented. In Table 2, we present the number of plants used in the wooden bowl-making process mentioned by people of different occupations. Among them, there are nine species used as wood materials, three species used to make dyes, and four species used to make varnishes. It is worth noting that the four *Impatiens* species have only one vernacular name, which may be related to the use of the *Impatiens* seeds. These surveys demonstrate the diversity of plants utilized in the process of making wooden bowls and also reflect the complexity of wooden bowl making in Gyirong Town.

**Table 2:**
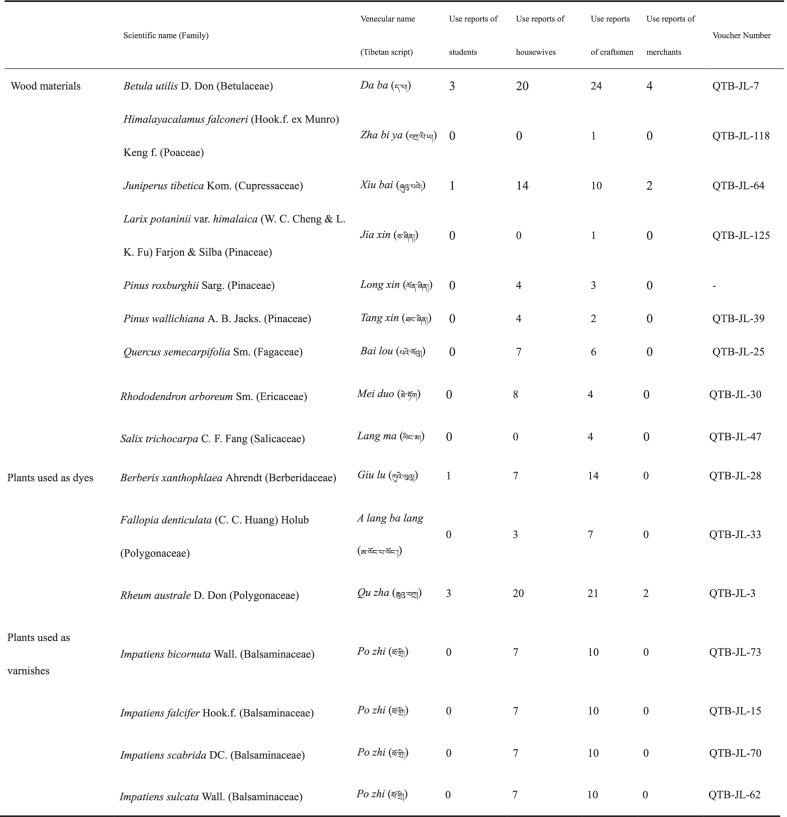
Plants used in the different stages for the manufacture of wooden bowls in Gyirong Town, Tibet, China

According to Table 2, we described the plants used in the making of wooden bowls mentioned by people of different occupations. *Betula utilis* D. Don, *Juniperus tibetica* Kom., and *Rheum australe* D. Don were mentioned by the informants of the four occupations. *Berberis xanthophlaea* Ahrendt was mentioned by one student but not by the merchant. This may be because the students were involved in the process when their parents used the plant to dye yellow. And craftsmen have no access to this process. Apart from the four plants described above, the students and merchants did not provide information on any other plants used in the making of the wooden bowls. The reason may be that (1) these four kinds of plants are the most commonly used plants in making wooden bowls, so they are well known by everyone, even if they are not professional wooden bowl craftsmen; (2) students and merchants are rarely involved in the making of wooden bowls. For plants as wood materials, *Himalayacalamus falconeri* (Hook.f. ex Munro) Keng f., *Larix potaninii* var. *himalaica* (W. C. Cheng & L. K. Fu) Farjon & Silba, and *Salix trichocarpa* C. F. Fang were mentioned only by craftsmen. For plants as dyes and varnishes, both housewives and craftsmen mentioned them. The reason may be that the craftsmen are the main finishers in the whole process of making the wooden bowls, and the housewives are the auxiliary roles. Therefore, the relevant traditional knowledge about making wooden bowls is mainly concentrated on craftsmen and housewives. Students and merchants know only common plant species.

### Plants as a source of wood

Through our interviews and fieldworks, we identified nine plant species (eight family and nine genera) used as wood for making wooden bowls in Gyirong Town (Table 2). All of these species were the dominant local vegetation types, indicating the sufficient availability of raw material resources. Due to the diversity of the wood nature of plants, they are chosen by people for different purposes [10]. The quality of the wooden bowls produced using the different plants varies, with local people showing some particular preference. The URs of different occupations reflect the local people’s understanding of different species of wood (Table 2). For example, *Betula utilis* was mentioned by people of all occupations because its wood does not crack or discolor, and the more that *B. utilis* bowls are used, the redder their color becomes. In contrast, bowl made using *Rhododendron arboreum*, which was mentioned only by housewives and craftsmen easily, deform over time. Because of the hardness of its wood, *Quercus semecarpifolia*, which was also mentioned only by housewives and craftsmen, is relatively difficult to work with, and the resulting bowls easily crack (Table 2). Due to the rarity of wooden bowls made by *H. falconeri*, a bowl can fetch around $3000. Also such differences between species, wooden bowls made from different parts of the same plant, can have different properties, which is reflected in their monetary value. For example, a bowl made from wood obtained from a tree trunk costs approximately $10, while a bowl made from burl wood can sell for $200–400. Indeed, burls are considered the best source of wood for bowl making by local Tibetans as these tend to produce items with the most beautiful patterns. But the value of wooden bowls made of burls from various trees is also different. The bowls made of *B. utilis* burls are the best and can sell for the highest price. Bowls made of *B. utilis* burls are called “cha-bo-luo” meaning they can last a lifetime; “cha-bo-luo” bowls are considered the best dowry and betrothal gifts, cherished carefully look after by their recipients. These specific types of bowls are also one of the most valued blessings passed between parents and their children for a happy life (Table 3).

**Table 3:** Plant part and wood characteristics of the species used as a source of wood in the manufacture of wooden bowls in Gyirong Town, Tibet, China

Scientific name (Family)	Use parts	Wood characteristics	Special purpose
*Betula utilis* D. Don (Betulaceae)	Stem and burl	The wood does not crack and firmly colored. And the pattern of wood is the most beautiful. Bowls made from this wood are the smoothest	Bowls made of burl are called “cha-bo-luo” meaning they can last a lifetime; “cha-bo-luo” bowls are considered the best dowry and betrothal gifts, cherished carefully look after by their recipients
*Himalayacalamus falconeri* (Hook.f. ex Munro) Keng f. (Poaceae)	Stalk base	The wood is rare	–
*Juniperus tibetica* Kom. (Cupressaceae)	Burl	This wood must have burls, and wood without burls cannot be used for bowls. And the wood should not be too dry, the wooden bowl made of too dry wood will crack	–
*Larix potaninii* var. *himalaica* (W. C. Cheng & L. K. Fu) Farjon & Silba (Pinaceae)	Burl	This wood must have burls, and wood without burls cannot be used for bowls	–
*Pinus roxburghii* Sarg. (Pinaceae)	Burl	This wood must have burls, and wood without burls cannot be used for bowls	–
*Pinus wallichiana* A. B. Jacks. (Pinaceae)	Burl	This wood must have burls, and wood without burls cannot be used for bowls	–
*Quercus semecarpifolia* Sm. (Fagaceae)	Stem and burl	The wood is too hard and easily crack	–
*Rhododendron arboreum* Sm. (Ericaceae)	Stem and burl	The wood is easily deform over time	–
*Salix trichocarpa* C*.* F. Fang (Salicaceae)	Burl	This wood must have burls, and wood without burls cannot be used for bowls	–

### Plants used as a source of dyes

**Fig. 2:**
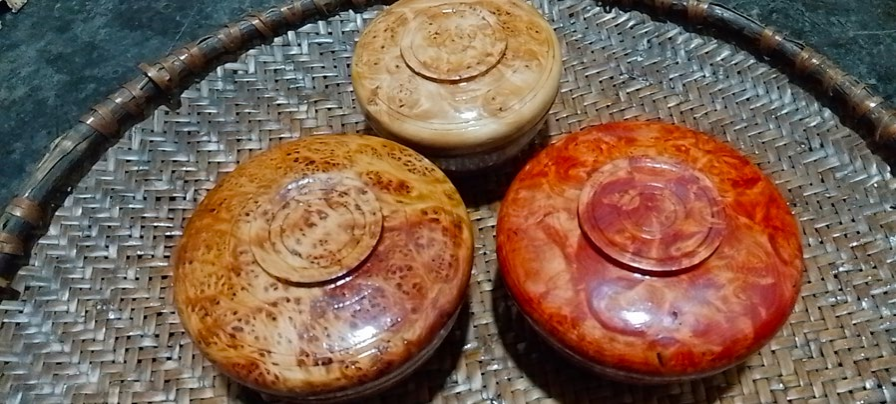
Wooden bowls of different colors in Gyirong Town, Tibet, China. The three bowls are made from wood of *B. utilis*. The top bowl is the undyed bowl, the left one is yellow dye (*R. australe*), and the right one is red dye (*R. australe* and *Fallopia denticulata*). The three bowls are varnished with industrial varnish

In Tibet, wooden bowls are dyed in yellow and red, having the meanings of good luck and happiness, respectively (Fig. 2). In Tibetan Buddhism, yellow represents the flourishing land, and red often represents fire as well as being a symbol of power. Typically, the *Rheum australe* rhizomes are used by locals in Gyirong to produce yellow dyes. And the barks or roots of *Berberis xanthophlaea* can also be used to produce yellow dyes (Table 4). First, the *R. australe* rhizomes are harvested from the fields, then the hull of the rhizomes is removed, and the remaining parts are directly used or sun-dried for later use (because these plants are often used for dyeing, it is too troublesome to collect if they are used). When you need to make dye solution, put fresh or dried rhizomes into boiling water about 30 min. After the dye solution has cooled, place the unfinished wooden bowls completely in the dye solution for about 30 s. Finally, the dyed bowl needs to be sun-dried. The process of dyeing yellow using the barks or roots of *B. xanthophlaea* is the same as for *R. australe*. Moreover, the used dye liquor should be kept for next use. If the concentration of the dye liquor next time is not enough, the corresponding plants can be added to increase the concentration. There are differences in the importance of plants that are used to dye the same color. Locals prefer to use *R. australe* which was mentioned by people of all occupations to dye yellow rather than *B. xanthophlaea* (in addition to craftsmen, people in other occupations mention this plant) (Table 2). The reason is because the local people think that the yellow dyed by *R. australe* is mixed with a faint red, while the yellow dyed by *B. xanthophlaea* is pure yellow. They prefer colors mixed with a faint red. Rhizomes of *R. australe* and *Fallopia denticulata* are used together to make red dye solution in the same way (Table 4). The concentration of the dye solutions depends on the type and color of the wood. For example, some wood (*B. utilis*) is “white”, requiring a higher concentration dye solution, while some types of wood are already “dark” in color and require a lighter dye solution. Notably, we made the first observation of *F. denticulata* being used for producing a dye. While traditional wooden bowl makers still use these dying methods, many others now use industrial dyes. In Gyirong, the yellow color of traditional Tibetan wooden bowl is still achieved using plant-based dyes, surviving as a deeply rooted cultural practice.

**Table 4:** Part of the plant, preparation and application of the species traditionally used for dying and varnishing wooden bowls in Gyirong Town, Tibet, China

	Scientific name (Family)	Use parts	Use and preparation
Plants used as dyes	*Berberis xanthophlaea* Ahrendt (Berberidaceae)	Bark and root	The barks or roots can also be used to produce yellow dyes. The dye solution preparation method is the same way as *Rheum australe*
	*Fallopia denticulata* (C. C. Huang) Holub (Polygonaceae)	Rhizome	Rhizomes of *Rheum australe* and *Fallopia denticulata* are used together to make red dye solution in the same way as *Rheum australe*
	*Rheum australe* D. Don (Polygonaceae)	Rhizome	The rhizomes are used to produce yellow dye solution. First, put dried rhizomes into boiling water about 30 min. After the dye solution has cooled, which can be used to dye yellow. And rhizomes of *Rheum australe* and *Fallopia denticulata* are used together to make red dye solution in the same way as *Rheum australe*
Plants used as varnishes	*Impatiens bicornuta* Wall. (Balsaminaceae)	Seed	The seeds are used to produce natural varnishes. First, collect mature *Impatiens* seeds, dry them, grind them, and coat the pre-dyed wooden bowls with the resulting oils
	*Impatiens falcifer* Hook.f. (Balsaminaceae)	Seed	
	*Impatiens scabrida* DC. (Balsaminaceae)	Seed	
	*Impatiens sulcata* Wall. (Balsaminaceae)	Seed	

### Plants used as varnishes

The term varnish refers to “a liquid that dries into a transparent film when applied to a solid surface” [23]. Wood varnishes not only protect the wood from weathering, abrasion, and environmental humidity fluctuations, but also have a certain decorative aesthetic function [23, 24]. In Gyirong, *Impatiens falcifer*, *I. bicornuta*, *I. sulcata*, and *I. scabrida* are used by locals to produce natural varnishes or lacquers (Table 4). These species were first documented to be used as varnishes. Of which the leaves of *I. sulcata* are edible, and the seeds can be eaten raw and processed into vegetable oils. Moreover, it is also used medicinally [25]. *I. scabrida* has medicinal and environmental uses [25]. At the beginning of October each year, the local people collect mature *Impatiens* spp. seeds, dry them, grind them, and coat the pre-dyed wooden bowls with the resulting oils. There was no difference in the utilization of the seeds of the four species of *Impatiens*, either separately or together. This process needs to be repeated at least three or four times. A similar process is reported to have been used for more than 7000 years in Chinese culture [26, 27]. China usually uses lacquer produced by *T. vernicifluum* to protect traditional wooden handicrafts [14]. The traditional wooden bowl lacquers used in the Sichuan Province, Yunnan Province, and Tibet region of China mainly consist of raw lacquer and tung oil purchased from the surrounding *Lisu* people [1]. Although lacquer-containing *T. acuminatum* and *T. wallichii* are found around the Gyirong Valley, they are not used by the locals [28]. This may be because local people wish to highlight the yellow and red dyes applied to their traditional bowls, and “raw lacquer” has quite a deep color; the natural dyes can be obscured by the color of the raw lacquer, with ancient Chinese lacquer mostly black and vermilion [29]. The unsaturated fatty acids content of *I. balsamina* seed oil is 70.75%, including 16.50% linoleic acid and 31.47% α-linolenic acid [30]. These substances penetrate into the wood, having a moisturize a preservative effect that highlights natural texture similar to Wood Wax Oil, which is 90% unsaturated fatty acids [31, 32]. Therefore, *Impatiens* varnish or lacquer is favored over that produced from *Toxicodendron* by the traditional wooden bowl makers in Gyirong.

### Process of the production of the bowls

**Fig. 3:**
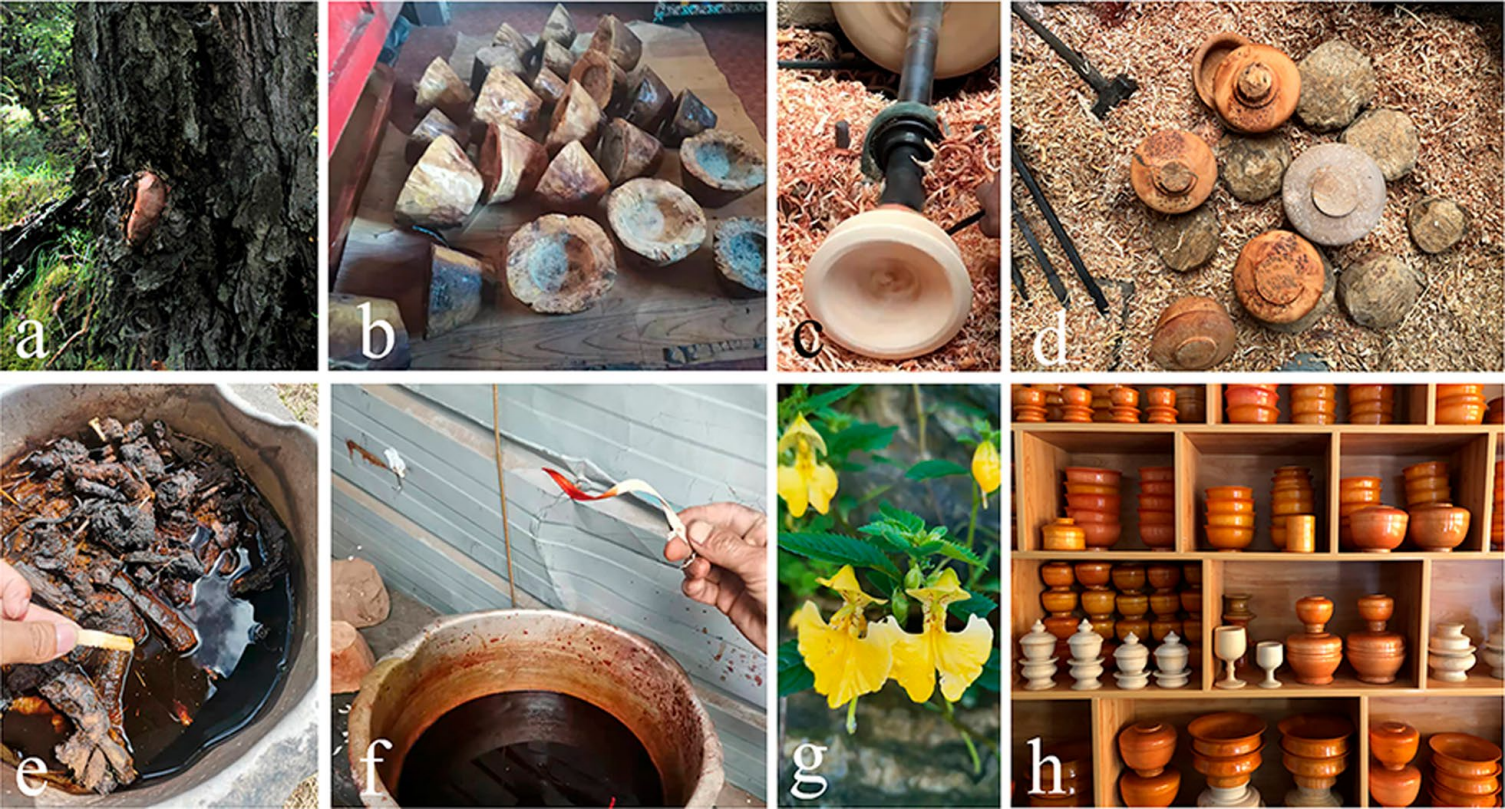
Processing of wooden bowls in Gyirong Town, Tibet, China. **a** Raw materials (the picture shows a burl of *Betula utilis* D. Don, Betulaceae); **b** Unfinished bowl; **c** machine processing; **d** rough wooden bowl; **e** yellow dye solutions from *Rheum australe*; **f** red dye solutions from *R. australe* and *Fallopia denticulata*; **g** traditional lacquer plant (*Impatiens falcifer* Hook.f., Balsaminaceae); and **h** finished wooden bowls

**Fig. 4:**
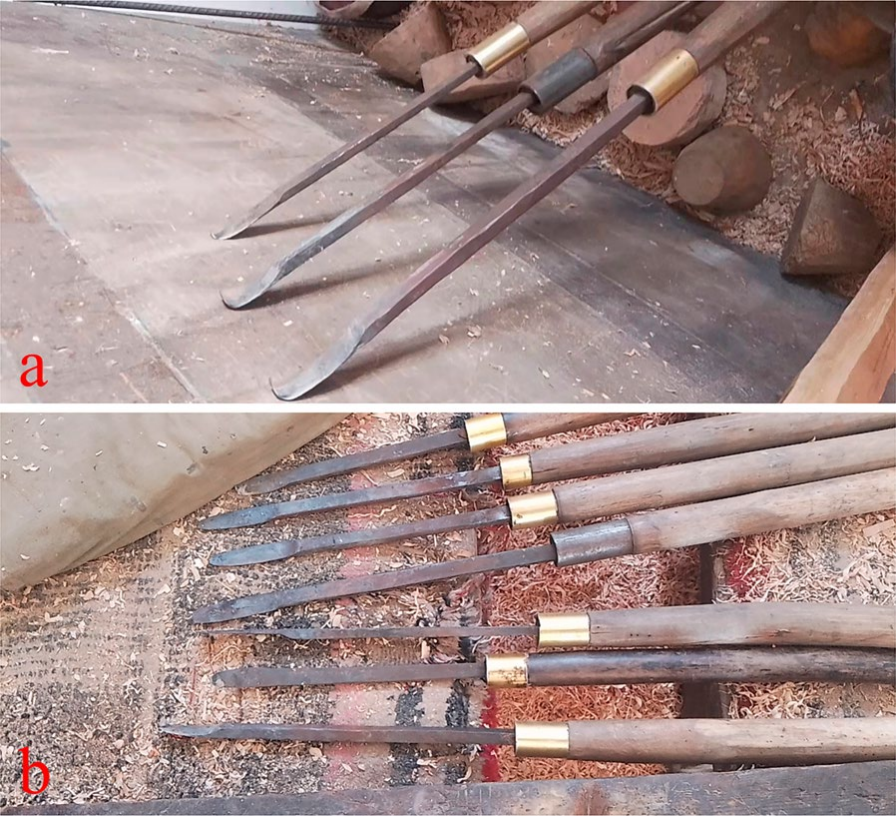
Tools for processing wooden bowls in Gyirong Town, Tibet, China. **a** The tools that used to work the inside of wooden bowls; **b** used for outside of the bowls

The wooden bowl production process is complicated, involving a series of steps: including material selection, airing, carving, dying, and varnishing (Fig. 3). According to our interviews and observations, it took several days to select the materials (Fig. 3a), and the raw materials required two or three months of airing before being used. Family members or relatives accompany wooden bowl-maker up the mountain to select and collect wood materials. The selected wood is then cut into a bowl shape with a knife. Rapeseed oil should be brushed over the bowl embryo. And it must also be placed in a place without direct sunlight but with adequate ventilation for a period of time in order to volatilize the moisture from the wood (Fig. 3b). The purpose is to prevent the bowl embryo from cracking. After that, rough processing is required. It takes about four hours to make a bowl (Fig. 3c, d). The most important part is the machining process, which necessitates the use of a variety of engraving tools. To begin, use a knife with a wider blade to trim the bowl embryo into the shape of a bowl, while the wood turner is rotating and then using a thinner blade to trim the details (Fig. 4). The knife used on the outside of the bowl is not the same as the one used on the inside (Fig. 4). Then, depending on different demands, dye the bowl in various colors (Fig. 3e, f). After dyeing, it must be sun-dried, and the surface of the bowl must be smoothed with sandpaper. Finally, grind the sun-dried *Impatiens* seeds and spread them on the bowl’s surface, repeating this process at least 3–4 times per bowl (Fig. 3e). The purpose of this process is to protect the bowl while also bring out the color and texture of the wooden bowl. In the end, a wide variety of wooden products is completed (Fig. 3h). Of course, certain wooden bowls can be used without being dyed or varnished.

### Harvesting practices for wooden bowl making and local management

Non-timber forest products provide a variety of cultural and economic values for those ethnic groups that depend on forests [22, 33]. However, whether target species for cultural and economic use can be managed sustainably remains a matter of debate. This includes *Juniperus Tibetica* (VU), which the International Union for Conservation of Nature (IUCN) has classified as a vulnerable species [34]. During our interviews, we learned about the forest management practices of the local people, which follow a general set of collection guidelines for *Betula utilis*, the most-used species in the area. Notably, local people believe that dead trees are the best raw material. For example, an earthquake that occurred on April 25, 2014, destroyed many *B. utilis* that were subsequently used to make wooden bowls. Harvesting time is also crucial, with the wood collected before the rainy season to preserve the valued patterns in the wood. These practices can be thought of as a sustainable management in Gyirong, much like “thinning” in other managed forests [35]. The impact of tree plants that used as handicraft materials to their populations may be low, because such activities are traditionally selective [10]. For other trees, locals tend to use wood with Burls, because the trunks of these trees make poor bowls. Also, removing the burls will not affect the growth of the trees. Furthermore, in recent years, the local government has stipulated that it is forbidden to fell certain tree species, including *J. tibetica*, and others can only be cut down three days of these year. This policy is generally implemented by the Chinese government throughout the country in order to protect forests [10]. During the interviews, the informant also reported that *J. tibetica* was less commonly used to process wooden bowls compared to before.

For dyed plants, the parts utilized (roots, tubers, bark) are all important parts of plant growth. Usually, locals go to the mountains to collect these plants after the leaves have fallen off. In their conception, the leaves absorb nutrients from the roots or rhizomes. After the leaves fall off, nutrients gather on the roots or rhizomes, and the dyed color is better. Also, current-year plants are usually not on their radar. Therefore, we speculate that this process has little impact on the natural population of the three dyed plants. Because the seeds of these plants have been dispersed after the leaves have fallen, the current-year plants are also preserved. According to our field observations, the number of wild individuals of these three plants is relatively abundant. However, the specific effect needs further experimental verification. The part of plant used as varnishes is seed. The fruit of *Impatiens* is a fleshy, explosive capsule; seeds often dispersed elastically from valves when ripe. Therefore, some seeds “escape” when they are collected. There were many *Impatiens* plants around the house. We also observed very rich natural populations during field work. But as local people use the seeds more and more, the impact on the natural populations of these plants requires further observation.

### Changes of varnish plants and wooden bowl handicraft industry respond to the market strategy

Traditional knowledge is often holistic and adaptive, gathered by generations of observers whose lives depend on this information and its use [36]. As an embodiment of the relationship between humans and biota, plant utilization fully reflects and supports the characteristics of culture, ideology and technology that existed at any given time in human history [37]. The process of re-selection and use of *Impatiens* plants by the Tibetans of Gyirong are a good example of this relationship. In the past, Gyirong wooden bowls were mainly produced in Naixia Village and were used by Tibetans in nearby towns and villages. With the development of society and tourism, Gyirong wooden bowls have become well known for their high quality and low price, and their demand is increased rapidly as a consequence. Due to the low yields of *Impatien*s seeds and the complicated processing procedures, traditional natural lacquer has been gradually replaced by modern industrial varnishes for over the past 30 years. However, commercial varnishes have one serious disadvantage. Although these varnishes can be easily purchased and application is relatively simple, they retain a pungent smell for a long period, which can have some health risks. In Gyirong, attempts are made to remove this smell by soaking the varnished wooden bowls in wine made from highland barley for half an hour, although this does not completely remove the smell. Perhaps most crucially, varnishing wooden bowls with commercial varnishes do not appear to improve sales. In contrast, a wooden bowl cooperative has been recently organized by the local people to promote the use of the original *Impatiens*-based wood varnish. This has been favored by tourists, and sales have increased significantly as a result both in self-operated and online stores. As such, *Impatiens* species in this area are more than just a traditional natural varnish or lacquer plant, but demonstrate the ability of local Tibetans to respond and adapt to the market strategy. Such response and adaptation rely on locally accumulated traditional knowledge [38].

In addition, according to the local craftsmen, there has been poor demand for ordinary wooden bowls over the last few decades, with generally poor profits as a result. Fewer local people make these items as a result, with many more choosing to seek work elsewhere. In the past, wooden bowls were only the processes in Nai village. Then, in 2008, the traditional wooden bowl-making process was selected, under the organization of the Gyirong Government, to be included on the Tibet Autonomous Region Intangible Cultural Heritage List [17]. At the same time, some people in other villages began to learn traditional techniques from their elders and more began to process and sell wooden bowls. More recently, the Gyirong Town Government has helped farmers and herdsmen register 51 professional cooperatives, including 12 processing and manufacturing cooperatives. This has helped spread traditional knowledge about wooden bowl making from Nai to other villages. In addition, the popularization of tourism and e-commerce has increased demand for these items. Villages with good tourism benefit from the wholesale of wooden bowls from Nai Village and Zha Village, further broadening the sales channels. Currently, craftsmen can earn up to $4000 each year from selling traditional wooden bowls, which are often made outside of the farming season. This additional income has become a powerful tool for local poverty alleviation.

Multiple challenges such as industrialization and globalization affect the development of handicraft industries worldwide [5, 39]. With the encouragement of the government, the local wooden bowl handicraft has shown a strong vitality in Gyirong Town. In addition, the local people in Gyirong are resilience to the rapid changes occurring around them, such as the use of four species of *Impatiens* for producing varnishes. Gyirong Town has rich forest resources. With the strong support of the government, the sustainable collection and utilization of wood bowl-related plants can promote the development of wood bowl handicraft industry and increase the income of local people.

## Conclusions

We studied the plants used in traditional Tibetan wooden bowl making in Gyirong Town, China, which included nine species belonging to nine genera. One of these species, *Juniperus tibetica* (VU), is identified as vulnerable by the IUCN. *Betula utilis* D. Don is the most commonly used species for bowl making because of its excellent characteristics. Bowls made from *B. utilis* burls are called “cha-bo-luo”, which are highly valued as dowry and betrothal gifts. The wooden bowls are dyed in yellow and red, signifying good luck and happiness, respectively. Yellow dyes are made from *Rheum australe* and *Berberis xanthophlaea*, and *R. australe* and *Fallopia denticulata* are used to make red dyes. *Impatiens falcifer*, *I. bicornuta*, *I. sulcata*, and *I. scabrida* are also used as varnish by the Tibetans in Gyirong. This is the first document of *F. convolvulus* being used to make red dye, and the first time these four species of *Impatiens* has been documented for use as wood varnishes.

We found that the wooden bowl craftsmen and their housewives have a rich traditional knowledge of using plants to make wooden bowls in Gyirong Town. Despite the fact that these plants are heavily relied upon by communities, good management and collection procedures were observed. The development of local plant resources can not only ensure a steady supply of raw materials for wooden bowls and maintain traditional handicraft industries, but also increase income. Notably, the continued use of *Impatiens* seed oils as varnishes over commercially available varnishes serves to demonstrate the resilience of these local communities and their traditional crafts. In the face of industrialization alongside the adaptability of local craftsmen and their wider communities, government support and guidance are highlighted as powerful tools to help protect and develop this fragile intangible cultural heritage. This case study provides inspiration for traditional handicrafts that are facing challenges elsewhere.

## Data Availability

Please contact the corresponding author for data requests.
